# Using the PowerMom Digital Health Platform to Support Prenatal Mental Health and Maternal Health Outcomes: Observational Cohort Study

**DOI:** 10.2196/70151

**Published:** 2025-05-22

**Authors:** Giulia Milan, Victoria Lee, Matteo Gadaleta, Lauren Ariniello, Arij Faksh, Giorgio Quer, Toluwalase Ajayi

**Affiliations:** 1 Scripps Research, La Jolla, CA, United States; 2 University of California San Diego, La Jolla, CA, United States; 3 The Dartmouth Institute, Lebanon, NH, United States; 4 Whoop Inc, Boston, MA, United States; 5 Scripps Perinatology, Scripps Clinic Medical Group, La Jolla, CA, United States; 6 Jacobs Center for Health Innovation, University of California, San Diego, 9300 Campus Point Drive, MC 7196, San Diego, CA, 92037, United States, 1 785-218-1643

**Keywords:** perinatal, mental health, postpartum depression, technology, mobile health

## Abstract

**Background:**

Mental health disorders such as anxiety and depression are common among individuals of childbearing age. Such disorders can affect pregnancy and postpartum well-being. This study aims to study the impact of prenatal mental health on the pregnancy journey and highlights the use of mobile health technologies such as PowerMom for symptom tracking and screening.

**Objectives:**

We collected data in a decentralized digital trial using the PowerMom platform to investigate the impact of maternal mental health throughout pregnancy. The goal was to understand how anxiety and depression influence pregnancy-related symptoms, pregnancy outcomes, and postpartum well-being.

**Methods:**

Survey data were collected via PowerMom, a bilingual mobile research platform that integrates patient-reported outcomes, wearable data, and electronic health records. Participants were divided into 2 cohorts: those who reported receiving treatment for anxiety or depression during pregnancy (n=571) and those who reported not receiving treatment (n=1505). We compared self-reported symptoms, prepregnancy conditions, complications from past pregnancies, delivery outcomes, and postpartum mental health between cohorts, using the Fisher exact test and the Kruskal-Wallis test for statistical analysis.

**Results:**

Participants receiving treatment for anxiety or depression reported higher instances of physical symptoms than those untreated, with significant differences for 13 symptoms including fatigue (80.2% vs 65.4%; adjusted *P*<.001), nausea and vomiting (69.7% vs 52.7%; adjusted *P*<.001), and stomach cramping and abdominal pain (64.0% vs 50.4%; adjusted *P*<.001). Participants receiving treatment also had a higher prevalence of several conditions prior to pregnancy than those not receiving treatment, with significant differences noted in 4 out of 10 conditions: endometriosis (14.0% vs 8.8%; adjusted *P*=.007), hypertension (10.9% vs 3.9%; adjusted *P*<.001), eating disorder (7.7% vs 3.1%; adjusted *P*<.001), and heart disease (2.8% vs 0.5%; adjusted *P*<.001). Participants receiving treatment also reported a higher prevalence of complications in past pregnancies than those not receiving treatment, with significant differences noted in 2 out of 7 complications: high blood pressure (9.9% vs 5.8%; adjusted *P*=.016) and preeclampsia (9.2% vs 5.5%; adjusted *P*=.021). No significant differences were observed in mode of delivery, epidural use, stillbirth, and miscarriage rates between the 2 cohorts. Postpartum, treated participants reported significantly higher mental health composite scores, indicating more severe mental health symptoms. A higher percentage of treated participants were at high risk for having perinatal mood disorder (38/83, 45.8%) than untreated participants (36/196, 18.4%; *P*<.001).

**Conclusions:**

The PowerMom platform demonstrated its value in facilitating remote, scalable data collection for maternal mental health research. Pregnant individuals reporting treatment for anxiety or depression experienced more physical symptoms and worse postpartum mental health outcomes than untreated individuals. These findings underscore the potential for mobile health technologies to support future interventional studies aimed at improving maternal mental health outcomes during pregnancy.

## Introduction

Mental health disorders such as anxiety and depression are common among individuals of childbearing age, impacting up to 1 in 5 women [1]. Research on mental health and pregnancy typically focuses on postpartum depression, a common condition that impacts 15%‐25% of those postpartum [2,3]. However, there is limited guidance on the ideal timing, frequency, and method for mental health screening before, during, and after pregnancy [4]. Some studies have identified prenatal depression as one of the strongest predictors of postpartum depressive symptoms, highlighting the importance of systematic screening and care coordination throughout pregnancy [5,6]. Similarly, antenatal anxiety has been associated with perinatal depression, underscoring the need to assess both conditions in research and clinical practice [6]. Nevertheless, studies exploring these issues remain scarce. There have been some limited data showing the association between prenatal mental health conditions and physical symptoms during pregnancy such as nausea and vomiting, as well as with delivery outcomes such as the use of epidurals and cesarean sections, but these also need further exploration [7,8].

We aim to expand this body of literature by using data from the PowerMom mobile research platform [9], which offers a novel digital framework to study and collect data from pregnant individuals from diverse backgrounds. PowerMom was chosen for this study because of its unique ability to facilitate bilingual, decentralized, and scalable data collection.

The platform collects longitudinal health data through participant-reported surveys and integrates psychological, social, and technological inputs related to maternal health. Participants have the option to connect wearable device data, allowing for a multidimensional view of their health throughout pregnancy. PowerMom’s decentralized nature enables remote participation, overcoming geographical barriers and expanding the reach to more diverse populations, thereby enhancing the generalizability of the study findings. Moreover, its bilingual capabilities allow participation from both English and Spanish speakers, improving inclusivity and engagement, particularly among underrepresented groups.

**Figure 1.**
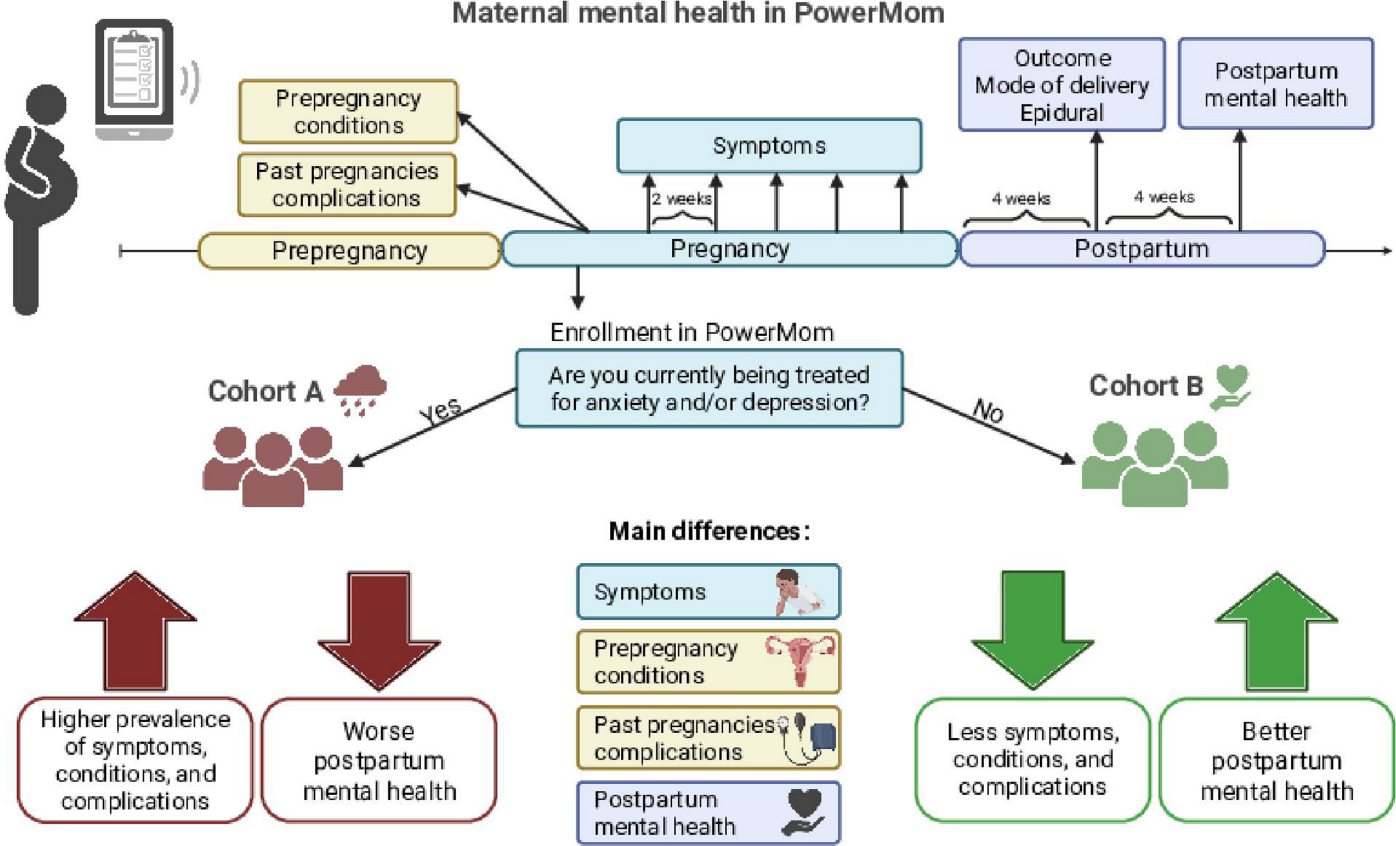
In PowerMom, participants enroll during pregnancy through their smartphone. At enrollment, they provide demographics information, due date, and answer surveys regarding current well-being, including whether they are being treated for anxiety or depression at that time. If yes, participants are included in cohort A (receiving treatment for anxiety and/ depression); otherwise, they are included in cohort B (not receiving treatment for anxiety or depression). Participants are then prompted to complete surveys regarding their health history, including questions about conditions diagnosed prepregnancy or complications experienced in past pregnancies. During their pregnancy, participants are prompted to complete biweekly surveys to track their pregnancy, such as physical symptoms experienced. Four weeks after their expected due date, participants are prompted with a delivery survey to report their pregnancy outcome, mode of delivery, and so forth. Around 8 weeks postpartum, participants are asked to complete a survey about their postpartum mental health. Participants in cohort A were more statistically significantly more likely to experience symptoms, to have been diagnosed with prepregnancy conditions, to have experienced complications in past pregnancies, and to have worse postpartum mental health than participants in cohort B. Created with BioRender.com.

By leveraging these features, this study examines the relationships among prenatal mental health, self-reported symptoms during pregnancy, preexisting conditions, complications from past pregnancies, delivery outcomes, and postpartum mental health, offering new insights into the interplay between mental health and pregnancy outcomes (Figure 1).

## Methods

### Study Design

In March 2021, The Scripps Research Institute expanded upon the Healthy Pregnancy study [10] and launched the PowerMom digital health study. This participant-centered research study is implemented as a bilingual (Spanish and English) mobile research platform.

Eligible participants include birthing people aged 16 years or older, currently pregnant or within 8 weeks postpartum, living in the United States and its territories, and with access to a smartphone. Participants are recruited via direct-to-participant methodologies including public announcements, social media outlets, and others. Those unable to read, speak, or consent in English or Spanish are excluded from the study.

Participants engage with PowerMom through surveys provided via the MyDataHelps app, with the option of sharing wearable device data with the platform. Wearable data, including heart rate, activity levels, and sleep patterns, were collected from participants who opted to sync their devices with the PowerMom platform. While not analyzed in this study, these objective measures will be examined in future research to complement and validate self-reported pregnancy symptoms and mental health outcomes. Surveys are presented to the participants at enrollment, during pregnancy, and postpartum. Data such as age, race and ethnicity, zip code, and pregnancy due date are reported by participants during study enrollment. Reported zip codes are used to determine the participants’ Area Deprivation Index (ADI) as defined by the Neighborhood Atlas, which is based on population-level data connecting geographical location to domains such as income, education, employment, and housing quality [11].

### Ethical Considerations

The PowerMom study was reviewed and approved by the Scripps Office for the Protection of Research Subjects (IRB-21‐7738). All participants provided informed consent electronically. Both the primary consent specify that the original consent and the institutional review board approval cover secondary analysis without additional consent. Data presented in this paper have been deidentified.

### Analytic Methods

#### PowerMom Survey Data

After consent, participants are asked to complete a series of surveys at preset intervals within the PowerMom research platform. (Methods 1—Participant Flow in Multimedia Appendix 1). After enrollment in the study, they complete a study intake survey to collect demographic information and a “Health and History” survey to provide details on their current and past health status. Throughout the course of their pregnancy, participants are asked to complete a biweekly “Health and Well-being” survey and a bimonthly “Pregnancy Support” survey. Based on self-reported due date, participants are alerted to complete the “Delivery” survey within 4 weeks of their due date and the “Postpartum” and “Postpartum Mental Health” surveys 6‐8 weeks after that date (Methods 2—Surveys in Multimedia Appendix 1).

##### Self-Reported Mental Health Diagnosis

Participants were asked whether they were currently receiving treatment for anxiety or depression at the time of enrollment in the “Health History” survey. The survey did not collect information on whether they had a prior history of mental health conditions before pregnancy. Cohort A includes participants who reported receiving treatment for anxiety or depression at the time of enrollment, and cohort B includes participants who reported not receiving treatment for anxiety or depression. It is important to note that this classification is based on treatment status rather than the presence or severity of symptoms. While cohort A consists of individuals receiving treatment for anxiety or depression, it does not necessarily include all individuals experiencing symptoms. Similarly, cohort B includes individuals who are not receiving treatment but may still have undiagnosed or untreated symptoms. Future research should incorporate validated symptom severity scales to better disentangle the effects of treatment from the presence of mental health conditions. Differences between the 2 cohorts were determined by analyzing additional survey responses during and postpregnancy, with participation varying by survey.

##### Self-Reported Symptoms During Pregnancy

Participants report symptoms in the biweekly “Health and Well-being” survey, noting any physical symptoms experienced in the previous 2 weeks by selecting them from a list, or selecting the “None of the above” option. The data capture symptom prevalence across both cohorts throughout pregnancy.

##### Self-Reported Past Conditions and Past Pregnancies Complications

In the “Health History” survey, participants are asked about any conditions diagnosed before their current pregnancy, accompanied by a selection list, including “Other” and “None of these” options. Participants are asked whether they experienced pregnancies in the past, whether they had any complications in their past pregnancy or pregnancies, and, if so, to select complications in past pregnancies with a list provided, including “Other” and “None of these” options. Participants who answered “Other” or “None of these” to these questions were excluded.

##### Pregnancy Outcomes

The “Delivery” survey includes questions on pregnancy completion. If the pregnancy concluded with a live birth, the participant is asked several follow-up questions about the mode of delivery, use of epidurals, whether delivery was induced, delivery date, and newborn-related information, such as weight and length. When available, due date, delivery date, and birth weight were used to evaluate the fraction of small for gestational age, appropriate for gestational age, or large for gestational age infants between the cohorts [12,13].

##### Postpartum Mental Health Symptoms

If participants did not report a miscarriage or pregnancy loss in the “Delivery” survey, they are prompted to complete the “Postpartum Mental Health” survey within 6‐8 weeks of their due date. The survey is used to evaluate the participant’s overall postpartum mental health through a composite mental health score. The survey and the composite score are based on the Edinburgh Postnatal Depression Scale. The Edinburgh Postnatal Depression Scale is a validated screening tool for perinatal mood disorders, with a cutoff score of ≥10 commonly used in clinical and research settings to identify individuals at risk. This threshold has demonstrated good sensitivity and specificity across diverse populations, although variations may exist based on cultural and demographic factors [14,15]. A score of 10 or greater is used to diagnose perinatal mood disorder. Patients with perinatal mood disorder are more likely to being diagnosed with postpartum depression. The survey includes 10 statements (eg, “I have been able to laugh and see the funny side of things”), with 4 possible responses for each statement (eg, “As much as I always could,” “Not quite as much,” “Definitely not so much now,” and “Not at all”), and a point value between 0 and 3 is assigned to each of the responses, with higher point values assigned to responses that signify worse mental health (Table S1 in Multimedia Appendix 1). Inclusion of this survey in our results allowed us to compare prenatal mental health by self-reported anxiety or depression with postpartum mental health scores.

### Statistical Analysis

#### Demographic and Baseline Characteristics

The demographics of participants, including age at enrollment, ADI, enrollment timing relative to conception, and gestational age at delivery, were reported as medians and the first and third IQRs (Q1, Q3). To account for potential confounding by socioeconomic status, we used the ADI, which provides a composite measure of socioeconomic disadvantage based on participants’ reported zip codes. The ADI was incorporated in our descriptive statistics and baseline cohort comparisons. While this approach offers an indirect measure of socioeconomic status, future analyses should incorporate additional individual-level metrics such as health care access and insurance status to further refine these findings. The Kruskal-Wallis test was used to determine statistical significance across the cohorts in terms of age, ADI, time of enrollment, and gestational age.

Race or ethnicity and preterm birth (delivery prior to 37 weeks of gestation) were expressed as count and percentage of participants among the ones answering the questions in the cohorts. The chi-square test was used for race or ethnicity and preterm births (Python package scipy; version 1.11.1; Python Software Foundation). Statistical significance was set at *P*<.05 for all results.

### Symptoms, Prepregnancy Conditions, and Past Pregnancy Complications

The “Health and Well-being” survey was administered multiple times during pregnancy. Symptom prevalence was defined as the percentages of participants reporting symptoms at least once across all survey responses. The “Health and History” surveys were administered only once. Prepregnancy conditions and past pregnancy complications were reported as percentages of participants reporting the conditions or complication in that survey. For our analysis, we reported all symptoms, prepregnancy conditions, and past pregnancy complications that were presented in the selection lists to the participants for each related survey question. The 95% CIs were calculated using the bootstrap bias-corrected and accelerated method with 10,000 iterations. Statistical significance was assessed using Fisher exact test (Python package FisherExact; version 1.4.2).

To mitigate the risk of increased false positives due to multiple comparisons, the Holm-Bonferroni correction was applied (Python package Pingouin; version 0.5.4) to compute adjusted *P* values (*P*_adj_) for an alpha significance at .05. This method was selected as it provides a balance between statistical power and control of type I error across multiple hypothesis tests. The correction was applied separately for each category of comparisons (symptoms, prepregnancy conditions, and past pregnancy complications) rather than across all tests combined, ensuring that each domain was adjusted independently. In total, 13 comparisons were corrected for assessing the differences in terms of symptoms between the 2 cohorts, 10 comparisons for prepregnancy conditions, and 8 comparisons were corrected for past pregnancy complications. No additional corrections were applied for multiple comparisons.

### Pregnancy Outcomes

For pregnancy outcomes, the percentage and 95% CIs between the 2 cohorts were computed using the bootstrap bias-corrected and accelerated method with 10,000 iterations. Overall significance between the cohorts was evaluated with either a multichoice or binary Fisher exact test.

### Postpartum

For postpartum analysis, the mean composite mental health score is computed for the 2 cohorts. The 95% CI is computed using the bootstrap bias-corrected and accelerated method with 10,000 iterations. Postpartum mental health scores differences were evaluated for statistical significance via the Kruskal-Wallis test. In addition, participants were assigned to 2 groups based on the postpartum mental health composite score: higher risk for perinatal mood disorder (score ≥10) or not (score <10) [14,15]. Binary hypothesis test with Fisher exact test was used to evaluate the statistical differences between the cohorts.

## Results

### Study Population

**Figure 2.**
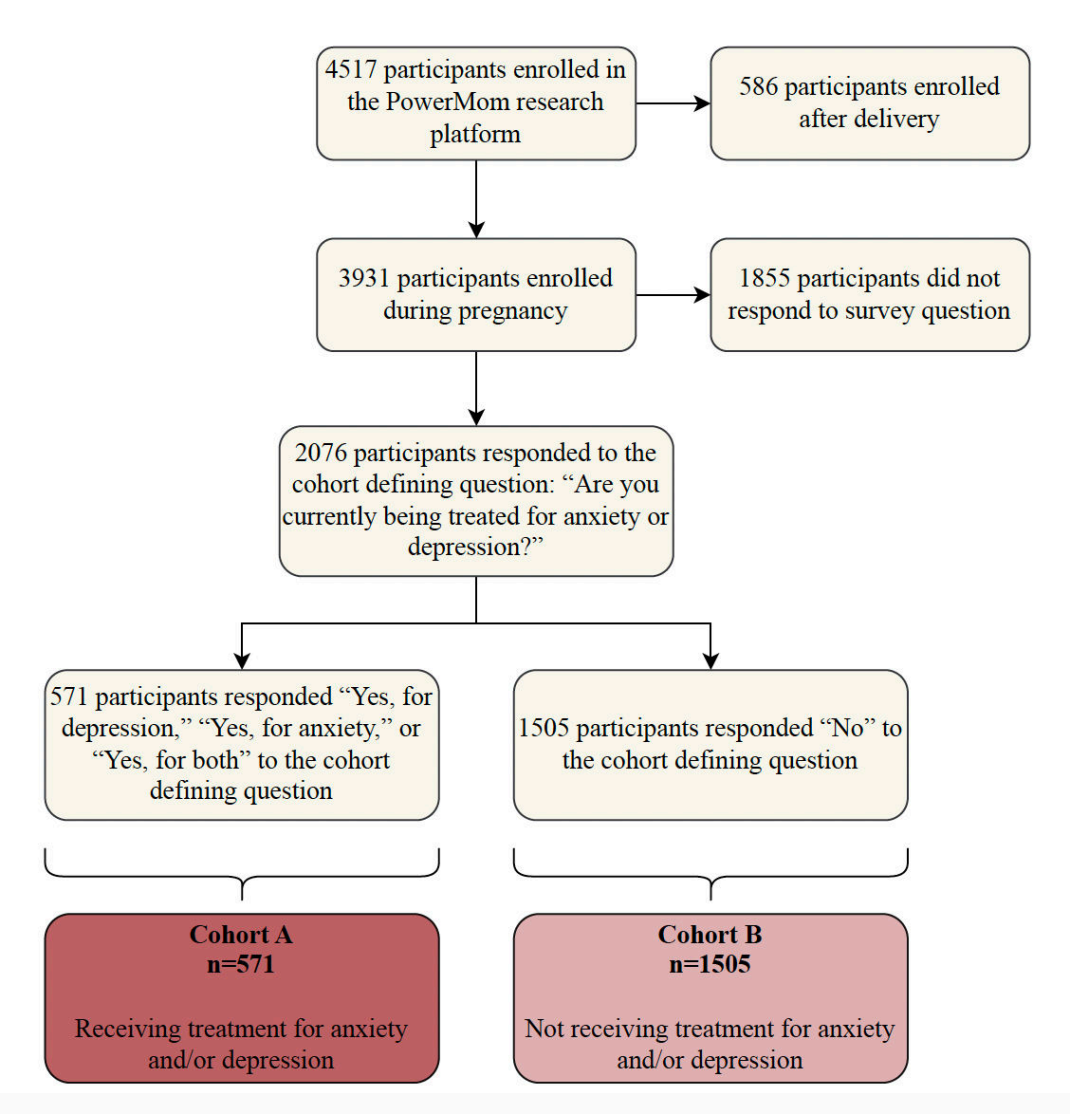
Participants inclusion diagram and segmentation into cohort A and cohort B. Cohort A (N=571) includes participants who responded “Yes, for depression,” “Yes, for anxiety,” or “Yes, for both” to the study intake survey question, “Are you currently being treated for anxiety or depression?”. Cohort B (N=1505) includes participants who responded “No.” Participants who did not respond to the survey question (N=1855) were excluded from this analysis.

Data collection through the PowerMom platform spanned from September 15, 2021, to June 18, 2024, with 4517 participants initially enrolled. Of these, 586 participants who enrolled postdelivery were excluded, leaving 3931 participants for analysis. From those, 2076 participants responded to the “Health History Survey” about anxiety or depression treatment: “Are you currently being treated for anxiety or depression?” The 571 participants who answered “Yes, for depression” (n=76), “Yes, for anxiety” (n=132), and “Yes, for both” (n=363) were included in cohort A. A total of 1505 participants responded “No” to the cohort-defining question and were included in cohort B. A total of 1855 participants did not respond to this survey question and were excluded from the analysis (Figure 2).

Differences in race and ethnicity between cohort A and cohort B were statistically significant (*P*<.001). A greater proportion of treated participants identified as White compared with those untreated (67.3% vs 49.7%), whereas a smaller proportion of treated individuals identified as Black or African American compared with those reporting not receiving treatment (8.8% vs 19.5%).

In addition, cohort A participants enrolled in the PowerMom study earlier in their pregnancy (*P*=.003), which might influence symptom reporting due to the frequency of survey administration. This early enrollment may impact the reliability of symptom data. To mitigate this issue, we included all participants who reported symptoms at least once through all the completed symptoms surveys.

The difference between the 2 cohorts in number of preterm births was not statistically significant. However, preterm birth information, including gestational age and delivery date, was available for a small fraction of the 2 cohorts (14.5% vs 14.6%). The 2 cohorts did not differ in terms of age at enrollment, ADI, gestational age, and birth weight (Table 1).

**Table 1.** Participants demographics of cohort A and cohort B^a^.

Characteristics	Cohort A (Receiving treatment for anxiety or depression at baseline; N=571)	Cohort B (Not receiving treatment for anxiety or depression at baseline; N=1505)	*P* value
Age (years), median (Q1,Q3)	32.0 (27.9,35.9)	32.0 (26.9,36.3)	.723
Provided, n (%)	502 (87.9)	1342 (89.2)	
Race and ethnicity, n (%)			
American Indian or Alaska Native	3 (0.6)	21 (1.5)	<.001^b^
Asian	8 (1.6)	39 (2.8)	
Black/African American	45 (8.8)	268 (19.5)	
Hispanic, Latino, or Spanish	38 (7.4)	169 (12.3)	
Middle Eastern or North African	0 (0.0)	7 (0.5)	
Multiracial	72 (14.0)	185 (13.4)	
Native Hawaiian or other Pacific Islander	2 (0.4)	3 (0.2)	
White	346 (67.3)	685 (49.7)	
Provided, n (%)	452 (88.8)	1171 (90.4)	
Neighborhood ADI^c^, median (Q1,Q3)	56.1 (37.3,72.8)	55.3 (32.7,73.3)	.253
Provided, n (%)	569 (99.6)	1489 (98.9)	
Enrollment relative to conception in days, median (Q1,Q3)	108.0 (63.0,182.0)	130.0 (70.0,195.0)	.003^b^
Provided, n (%)	515 (90.2)	1395 (92.7)	
Preterm births, n (%)	10 (12.0)	21 (9.6)	.677
Provided, n (%)	83 (14.5)	219 (14.6)	
Gestational age at delivery in weeks, median (Q1, Q3)	39.0 (37.5,39.9)	39.1 (38.1,39.9)	.143
Provided, n (%)	83 (14.5)	219 (14.6)	
Birth weight in grams, median (Q1, Q3)	3401.9 (2941.2,3685.4)	3260.2 (2976.7,3628.7)	.449
Provided, n (%)	84 (14.7)	198 (13.2)	

a Comparison of demographic characteristics for participants in cohort A (receiving treatment for anxiety or depression) and cohort B (not receiving treatment for anxiety or depression). Age, race or ethnicity, Area Deprivation Index, time of enrollment relative to conception, number of preterm births, gestational age, and birth weight across the 2 cohorts are shown. Preterm births were computed as delivery happening before completing 37 weeks of gestation. Chi-square and Kruskal-Wallis tests were used to determine statistically significant differences across the cohorts.

b Statistical significance at the .05 level.

c ADI: Area Deprivation Index.

### Self-Reported Symptoms During Pregnancy

**Figure 3.**
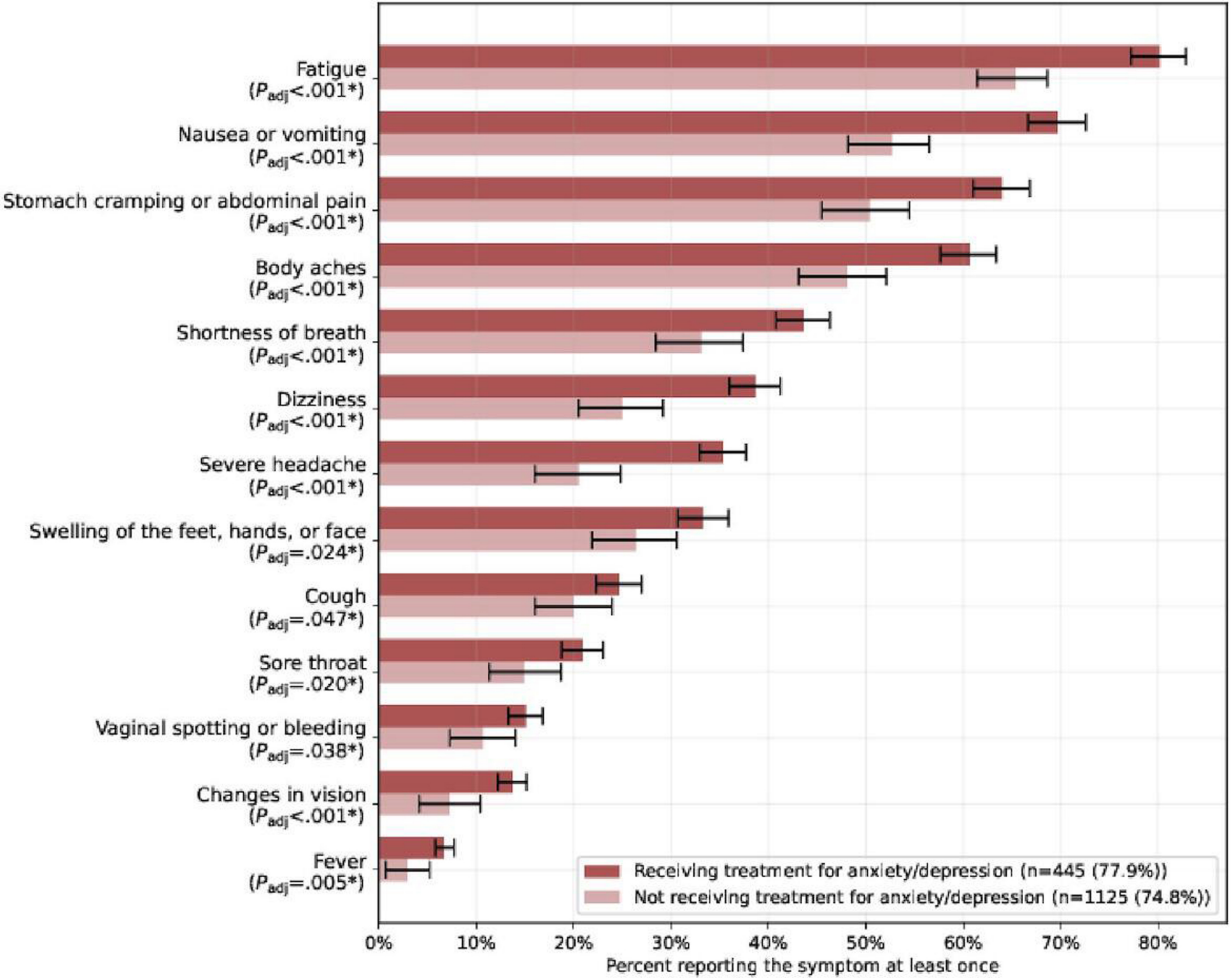
Physical symptoms reported during pregnancy by participants in cohort A and cohort B. The fraction of participants who answered the survey at least once in cohort A (receiving treatment for anxiety or depression) and cohort B (not receiving treatment for anxiety or depression) experiencing each symptom during pregnancy. *Statistical significance at the .05 level. The *P* value (*P*_adj_) is calculated with the Holm-Bonferroni correction.

Participants who reported receiving treatment for anxiety or depression (cohort A) were more likely to report experiencing physical symptoms across all tracked symptoms than untreated participants (cohort B). Applying the Holm-Bonferroni correction (significance at .05), the difference among participants experiencing a symptom across the 2 cohorts was statistically significantly higher for cohort A for all 13 listed symptoms: fatigue (80.2% vs 65.4%; *P*_adj_<.001); nausea or vomiting (69.7% vs 52.7%; *P*_adj_<.001); stomach cramping or abdominal pain (64.0% vs 50.4%; *P*_adj_<.001); body aches (60.7% vs 48.1%; *P*_adj_<.001); shortness of breath (43.6% vs 33.1%; *P*_adj_<.001); dizziness (38.7% vs 25.0%; *P*_adj_<.001); severe headache (35.3% vs 20.5%; *P*_adj_<.001); swelling of the feet, hands, or face (33.3% vs 26.4%; *P*_adj_=.024); cough (24.7% vs 20.0%; *P*_adj_=.047); sore throat (20.9% vs 14.9%; *P*_adj_=.020); vaginal spotting or bleeding (15.1% vs 10.7%; *P*_adj_=.038); changes in vision (13.7% vs 7.2%; *P*_adj_<.001); and fever (6.7% vs 2.9%; *P*_adj_=.005) (Figure 3 and Table S2 in Multimedia Appendix 1).

### Self-Reported Past Conditions and Past Pregnancies Complications

**Figure 4.**
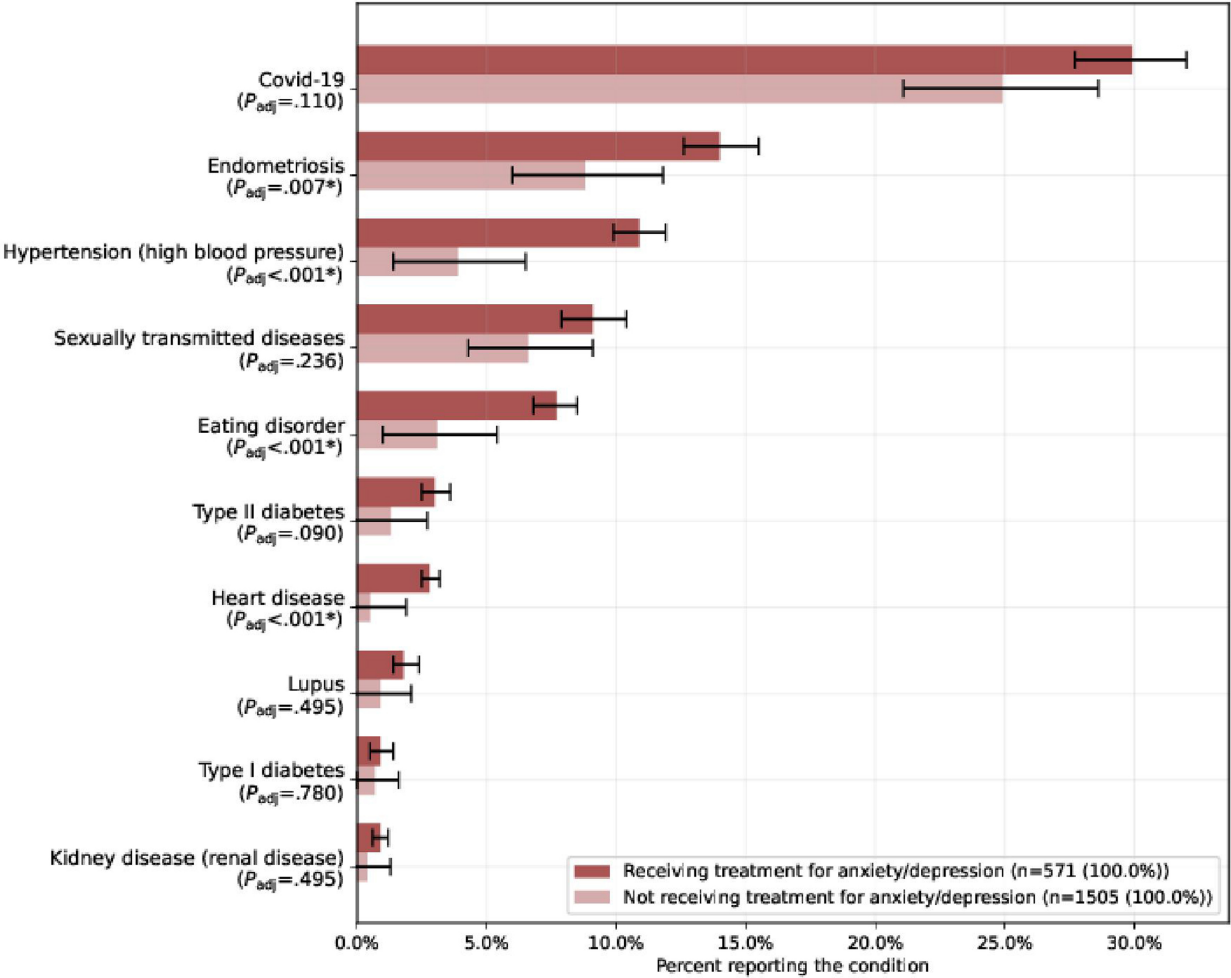
Conditions diagnosed prepregnancy by participants in cohort A and cohort B. The fraction of participants who answered the survey in cohort A (receiving treatment for anxiety or depression) and cohort B (not receiving treatment for anxiety or depression) who were diagnosed with such conditions before the current pregnancy. *Statistical significance at the .05 level. The *P* value (*P*_adj_) is calculated with the Holm-Bonferroni correction.

Treated participants (cohort A) were more likely to report preexisting medical conditions across all tracked conditions than untreated participants (cohort B). Applying the Holm-Bonferroni correction, the difference among participants diagnosed with a condition across the 2 cohorts was statistically significantly higher for cohort A for 4 out of 10 listed conditions: endometriosis (14.0% vs 8.8%; *P*_adj_=.007), hypertension (10.9% vs 3.9%; *P*_adj_<.001), eating disorder (7.7% vs 3.1%; *P*_adj_<.001), and heart disease (2.8% vs 0.5%; *P*_adj_<.001) (Figure 4 and Table S3 in Multimedia Appendix 1).

**Figure 5.**
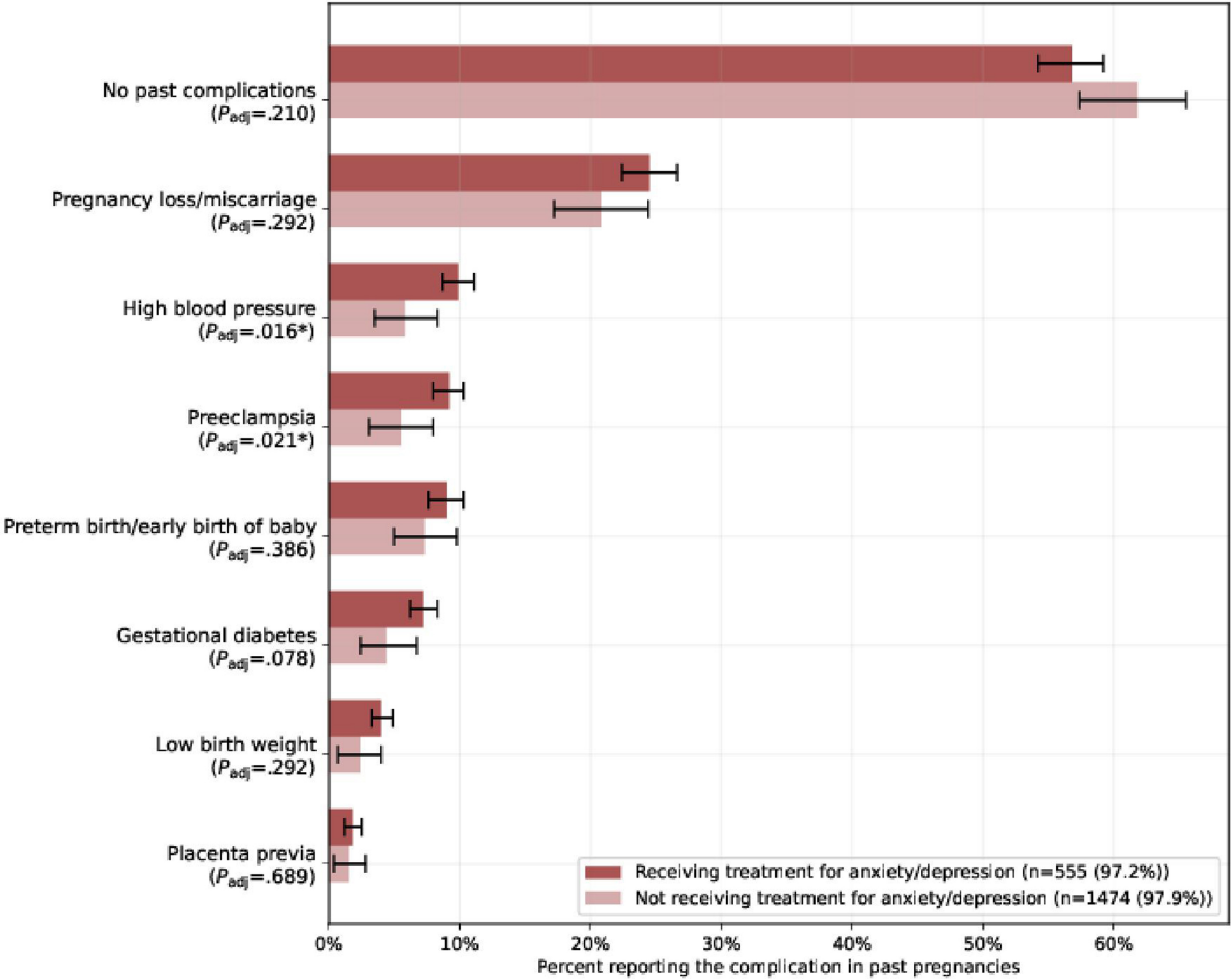
Reported complications in past pregnancies by participants in cohort A and cohort B. The fraction of participants who answered the survey from cohort A (receiving treatment for anxiety or depression) and cohort B (not receiving treatment for anxiety or depression) reporting experiencing each complication during past pregnancies. *Statistical significance at .05 level. The *P* value (*P*_adj_) is calculated with the Holm-Bonferroni correction.

Complications in prior pregnancies were more prevalent in cohort A across all tracked complications than in untreated participants (cohort B). Applying the correction, the difference among participants who experienced a complication in past pregnancies across the 2 cohorts was statistically significantly higher for cohort A for 2 out of 7 listed complications: high blood pressure (9.9% vs 5.8%; *P*_adj_=.016) and preeclampsia (9.2% vs 5.5%; *P*_adj_=.021) (Figure 5). Gestational diabetes was not statistically significant with the Holm-Bonferroni correction. Participants who had previous pregnancies but did not have complications in past pregnancies were more prevalent in cohort B than in cohort A; however, the difference was not significant with the correction. Results without the correction are available for all conditions and complications presented in the analysis (Table S4 in Multimedia Appendix 1).

### Pregnancy Outcomes

Treated participants (cohort A) were more likely to experience a miscarriage or a stillbirth (14.8% and 4.3%) than untreated participants of cohort B (11.3% and 1.5%). These differences were not significant (*P*=.142). A higher percentage of participants in cohort A (71/93, 76.3%) received an epidural than in cohort B (151/231, 65.4%); however, the difference was nonsignificant (*P*=.065). No differences were found between the 2 cohorts in terms of labor induction, mode of delivery, and neonatal size for gestational age. Fewer participants for each cohort completed the “Delivery” survey for pregnancy outcomes (cohort A: 115/571, 20.1%; cohort B: 265/1505, 17.6%) and delivery-related questions (cohort A: 93/570, 16.3%; cohort B: 231/1509, 15.3%) (Table S5 and Figure S1 in Multimedia Appendix 1). The response rate was much higher for the surveys on symptoms, prepregnancy conditions, and past pregnancies complications.

### Postpartum Mental Health Symptoms

The postpartum mental health composite score was significantly higher for treated participants (cohort A) than that for untreated participants (mean 9.94, 95% CI 8.37-11.63 vs 5.80, 95% CI 5.15-6.53; *P*<.001). The distribution of postpartum mental health composite scores for cohort A and cohort B is available (Figure S2 in Multimedia Appendix 1). Treated participants were at higher risk for perinatal mood disorder based on the survey than untreated participants (45.8% vs 18.4%; *P*<.001) (Multimedia Appendix 2).

## Discussion

### Principal Findings

We reported strong associations between self-reported prenatal mental health treatment and postpartum mental health outcomes. The significant correlation between prenatal and postpartum mental health, supported by a validated survey, underscores the critical importance of addressing prenatal mental health conditions. Although we did not observe statistically significant differences in pregnancy outcomes between the 2 cohorts, several factors may explain these findings. These include cohort heterogeneity, sample size limitations, or additional unmeasured variables influencing obstetric outcomes. These results align with prior research showing mixed evidence regarding the impact of prenatal anxiety and depression on delivery outcomes. Future studies with larger, more targeted cohorts could help clarify these relationships and identify potential moderating factors. Regardless, there were notable disparities in physical symptoms, preexisting conditions, and past pregnancy complications. These findings highlight the complex relationships between prenatal mental health and overall pregnancy experiences. While this study provides key insights into prenatal mental health and its associations with pregnancy and early postpartum outcomes, long-term follow-up was beyond its scope. Other research leveraging digital health platforms such as PowerMom could expand on these findings by incorporating longitudinal postpartum assessments to track mental health trajectories over time.

### Results in the Context of What Is Known

These findings enrich and expand upon existing research by elucidating the link between prenatal and postpartum mental health [2] and between prenatal mental health and pregnancy-related outcomes. Our longitudinal data reveal that individuals who reported being treated for anxiety or depression during pregnancy reported more physical symptoms, such as nausea and vomiting, consistent with some previous findings [8,16] and contrary to other findings that focused on the usage of antidepressants and physical symptoms [17]. This discrepancy highlights the need for further investigation into the complex relationships between mental health and physical symptoms during pregnancy.

Although differences in pregnancy outcomes between the 2 cohorts were not significant, data showed an increase of miscarriage, stillbirth outcomes, and use of epidural in participants treated for anxiety or depression, aligning our research to previous findings [5-7,18].

Treated individuals reported more complications and preexisting conditions, such as endometriosis, aligning with previous research on the impact of endometriosis on mental health [19]. Previous research showed how COVID-19 may negatively affect postpartum mental health [20], and how complications during pregnancy and at birth are associated with increased risk of postpartum depression, long-term depression, anxiety disorder, and posttraumatic stress disorder [21,22]. Postpartum depression may have harmful consequences on the birthing person and child [23]. Hence, depression should be detected and treated as early as possible [23], and complications and postpartum physical symptoms should be taken into account when screening for postpartum depression [21,22,24].

### Clinical Implications

As an observational study, our findings describe associations rather than establish causal relationships. While we observed significant differences in postpartum mental health outcomes between treated and untreated individuals, we cannot determine whether treatment itself influenced these outcomes or whether underlying differences in mental health severity, access to care, or other confounders contributed to the observed patterns. Future research should consider randomized or quasi-experimental study designs to better isolate treatment effects.

Our results validate how digital technology can be effectively used in mental health screening techniques. Current screening methods during pregnancy could be improved by including systematic assessment of physical symptoms and pregnancy outcomes.

In addition, we observed more participants identified as Black in the no-treatment cohort with respect to the treatment cohort, potentially reflecting racial and ethnic disparities in the treatment of anxiety and depression. This distribution supports known access to care issues [25-27] and raises critical questions about access and the influence of structural inequities and medical mistrust [28]. The interpretation of this result is indeed complicated by the fact that participants in cohort B reported fewer symptoms and complications. A further investigation into the intersection of race, treatment access, and health outcomes in pregnancy will be needed to properly interpret this finding.

### Research Implications

The use of the PowerMom platform exemplifies how decentralized trials and digital health technology can enhance participant engagement and data collection, extending the reach of maternal health research beyond traditional settings. This approach, demonstrated by our study’s alignment with previous findings, confirms the use of digital health platforms in broadening our understanding of maternal health dynamics, supporting the effectiveness of digital health platforms [29]. However, our results also highlight the need for careful consideration of equity in mental health care access. Digital platforms such as PowerMom, while effective for data collection, may exacerbate existing disparities in access to smartphone technology and health care. The digital divide may limit participation among lower-income or marginalized populations [30,31].

### Strengths and Limitations

A key strength of this study is the large, diverse sample collected through the PowerMom platform, which allowed us to analyze data across multiple dimensions of maternal health. By integrating these various dimensions, this study offers a comprehensive view of the participants’ health, going beyond traditional clinical health records. However, potential clinician bias in diagnosing and treating mental health conditions may have influenced which patients reported receiving treatment, reflecting broader systemic barriers to care for certain racial and ethnic groups [28]. Finally, this study successfully engages a diverse cohort, potentially increasing the generalizability of the findings and providing insights into populations often underrepresented in clinical research.

A key limitation is the reliance on self-reported treatment status, which varies widely from meditation to medication possibly affecting consistency in treatment classification. It is important to acknowledge that our classification was based on treatment status rather than the presence of symptoms alone. While treatment status during pregnancy was self-reported, we cannot determine whether participants had a history of anxiety or depression prior to conception, which may have influenced both their treatment decisions and symptom presentation. Furthermore, treatment status does not directly reflect symptom severity. Some participants in the untreated cohort may still experience significant anxiety or depression symptoms, while others in the treated cohort may have had milder conditions warranting intervention. This limitation may have introduced heterogeneity in the analysis and highlights the need for future studies to assess both treatment status and clinical symptom severity separately to improve interpretability. Participants undiagnosed or not being actively treated for anxiety or depression were not captured in this analysis. This reinforces the need for future research to explore confounding factors, such as whether participants’ anxiety and depression were driven by comorbid conditions, or vice versa. Furthermore, we found differences between the cohorts in terms of race and ethnicity. Future analyses should objectively assess the presence of these conditions and include covariates such as race and ethnicity to further evaluate the relationships between anxiety and depression treatment, race and ethnicity, and measured outcomes. Furthermore, while ADI was used as a proxy for socioeconomic status, it does not capture all individual-level factors influencing mental health and prenatal care quality. Future studies should explore additional confounders, such as direct measures of income, health care access, and social support networks, to enhance causal interpretations.

In addition, we did not report results for conditions identified during the current pregnancy via the “Health and Well-being” survey. While more than 70% of participants in each cohort completed the survey, the prevalence of specific conditions was low (eg, fewer than 4 participants reported preeclampsia), leading to not statistically significant results due to the small sample size. This limitation might stem from the timing of diagnosis, as many conditions manifest later in pregnancy, and not all participants completed the biweekly surveys up to delivery.

Relatedly, this low prevalence of certain conditions reported during the current pregnancy due to timing and incomplete survey responses, coupled with significant attrition as the study progressed, was a limitation.

### Conclusions

This study underscores the essential role of early and continuous mental health screenings for expectant individuals, emphasizing the association between prenatal mental health and various maternal outcomes. We found that those receiving treatment for anxiety or depression experienced more physical symptoms and poorer postpartum mental health. However, these findings reflect associations rather than causal relationships, and further interventional studies are necessary to establish causality. Significant disparities in treatment for prenatal mental health across racial groups call for targeted interventions to ensure equitable care. Our findings also demonstrate the potential of digital health technologies to enhance screening and research, laying the groundwork for future studies on the impacts of mental health conditions.

## Supplementary material

10.2196/70151 Multimedia Appendix 1 Participant flow, surveys, and results by cohort, including detailed survey instruments and analyses related to postpartum mental health outcomes, pregnancy symptoms, prepregnancy conditions, past pregnancy complications, and obstetric outcomes. Results compare 2 cohorts: cohort A (receiving treatment for anxiety or depression at baseline) and cohort B (not receiving treatment). Statistical significance was evaluated using Fisher exact test with Holm-Bonferroni correction applied. Figures display key obstetric outcomes and the distribution of composite postpartum mental health scores, based on the Edinburgh Postnatal Depression Scale.

10.2196/70151 Multimedia Appendix 2 Postpartum composite mental health scores for cohort A and cohort B. Severity of self-reported postpartum mental health symptoms for participants in cohort A (receiving treatment for anxiety or depression) compared with participants in cohort B (not receiving treatment for anxiety or depression). Postpartum composite mental health scores are determined using the scoring based on the Edinburgh Postnatal Depression Scale (EPDS) [14]. A score of 10 or higher indicates that the participant is at higher risk for perinatal mood disorder and for developing postpartum depression.

## Abbreviations

ADI: Area Deprivation Index
