# Supplementary material for: Using the PowerMom Digital Health Platform to Support Prenatal Mental Health and Maternal Health Outcomes: Observational Cohort Study
Source: JMIR Ment Health. 2025 May 22;12:e70151. doi: 10.2196/70151 (PMC12121545; doi:10.2196/70151)
Supplement: Multimedia Appendix 1 [file mental-v12-e70151-s001.docx]

**Supplementary Methods 1—Participant Flow**

**Supplementary Methods 2—Surveys**

**
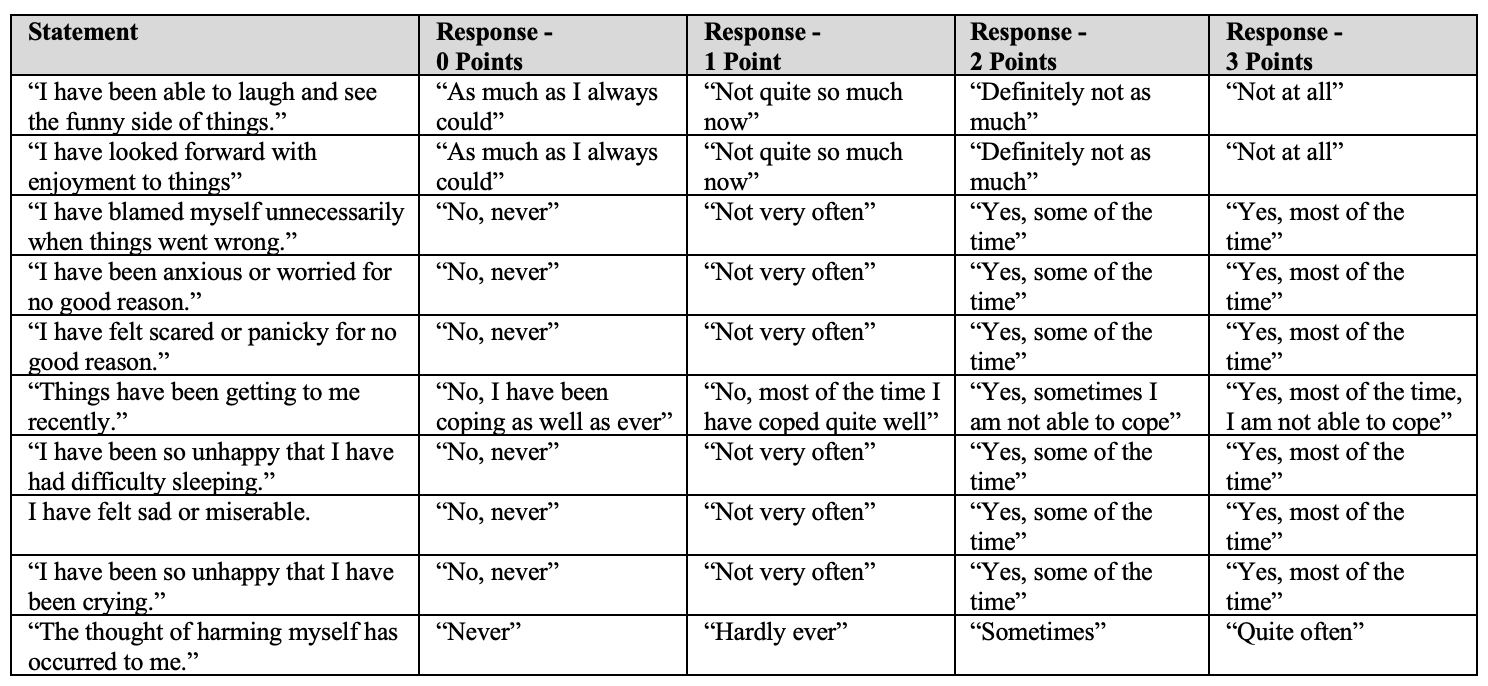
**

**Table S1. Postpartum Mental Health Survey Questions and Point Value for Composite Score.**

Survey statements for the postpartum mental health survey with the multiple-choice answers and assigned point values for composite postpartum mental health score, based on the Edinburgh Postnatal Depression Scale (EPDS). The composite score is calculated by summing the point values of all responses. A lower composite score corresponds to better postpartum mental health.

**Table S2. Symptoms reported during pregnancy by participants in Cohort A and Cohort B.**

The fraction of participants who answered the survey at least once in Cohort A (Receiving Treatment for Anxiety or Depression at Baseline) and Cohort B (Not Receiving Treatment for Anxiety or Depression at Baseline) experiencing each symptom during pregnancy. Fisher’s exact test was used to determine statistically significant differences across the cohorts in terms of p-value.

* Indicates statistical significance at the 0.05 level with the Holm-Bonferroni correction applied. Adjusted p-values are available in the manuscript.

**Table S3. Pre-pregnancy conditions reported by participants in Cohort A and Cohort B.**

The fraction of participants who answered the survey in Cohort A (Receiving Treatment for Anxiety or Depression at Baseline) and Cohort B (Not Receiving Treatment for Anxiety or Depression at Baseline) being diagnosed with each condition during pregnancy. Fisher’s exact test was used to determine statistically significant differences across the cohorts in terms of p-value.

* Indicates statistical significance at the 0.05 level with the Holm-Bonferroni correction applied. Adjusted p-values are available in the manuscript.

**Table S4. Past pregnancies complications** **reported by participants in Cohort A and Cohort B.**

The fraction of participants who answered the survey in Cohort A (Receiving Treatment for Anxiety or Depression at Baseline) and Cohort B (Not Receiving Treatment for Anxiety or Depression at Baseline) who experienced complications in past pregnancies. Fisher’s exact test was used to determine statistically significant differences across the cohorts in terms of p-value.

* Indicates statistical significance at the 0.05 level with the Holm-Bonferroni correction applied. Adjusted p-values are available in the manuscript.

**Table S5. Obstetric outcomes for participants in Cohort A and Cohort B.**

Differences between self-reported obstetric outcomes for participants in Cohort A (Receiving Treatment for Anxiety or Depression at Baseline) and Cohort B (Not Receiving Treatment for Anxiety or Depression at Baseline): pregnancy outcome, mode of delivery, epidural usage, induction of labor, and number of infants born small for gestational age (SGA), appropriate for gestational age (AGA), and large for gestational age (LGA). Preterm births were computed as delivery happening before completing 37 weeks of gestation, up to 258 days. The “Delivery” survey included questions regarding delivery date, and newborn information, such as weight. When available, due date, delivery date, and birthweight were used to evaluate the fraction of SGA, AGA, or LGA infants between the cohorts [1,2].

**
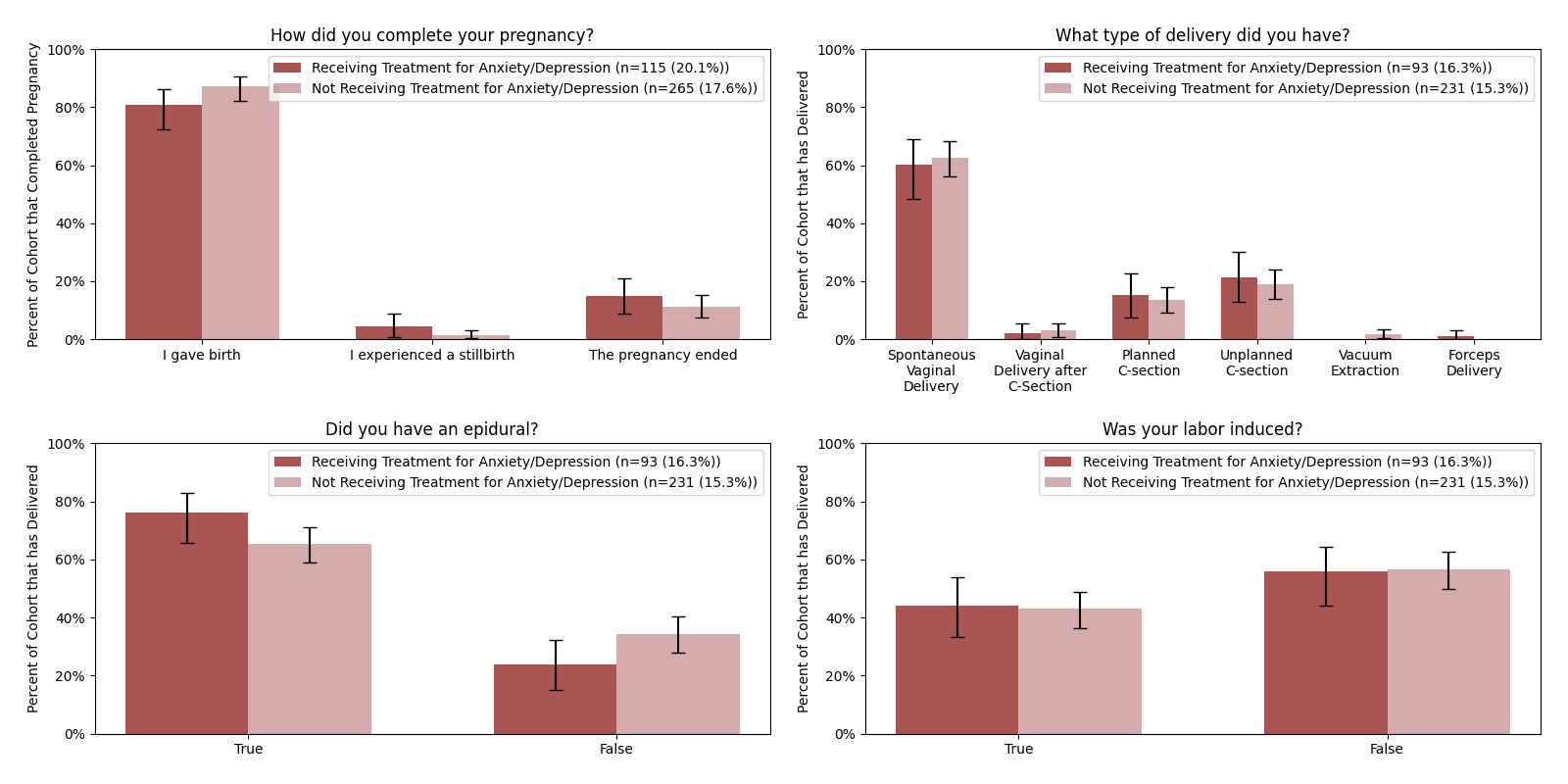
**

**Figure S1. Obstetric outcomes for participants in Cohort A and Cohort B.**

Differences in obstetric outcomes for participants on Cohort A (Receiving Treatment for Anxiety or Depression at Baseline) and Cohort B (Not Receiving Treatment for Anxiety or Depression at Baseline): pregnancy outcomes, mode of delivery, having received an epidural, and participants for which labor was induced.


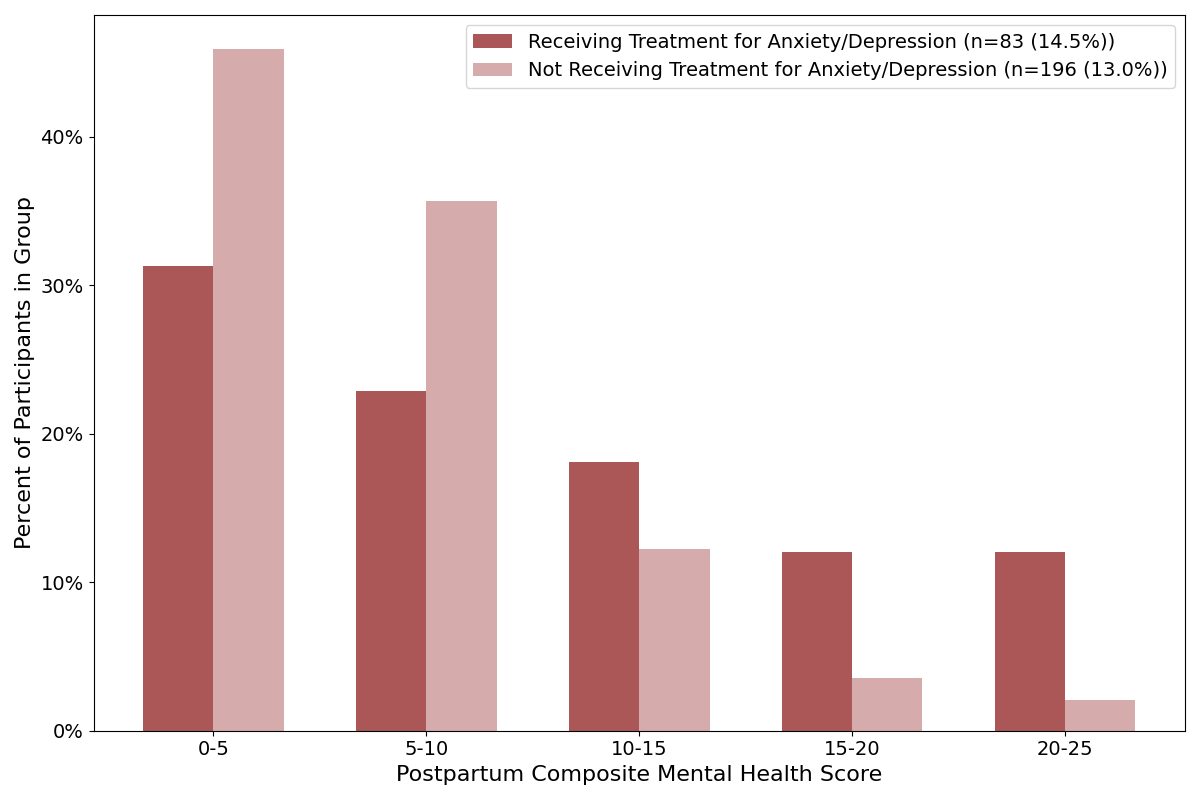


**Figure S2. Distribution of postpartum composite mental health scores for Cohort A and Cohort B.**

Severity of self-reported postpartum mental health symptoms for participants in Cohort A (Receiving Treatment for Anxiety or Depression at Baseline) compared to participants in Cohort B (Not Receiving Treatment for Anxiety or Depression at Baseline). Postpartum composite mental health scores are determined using the scoring based on the Edinburgh Postnatal Depression Scale (EPDS). A higher score indicates the participant reported more severe mental health symptoms.

### REFERENCES

1. Fenton TR, Kim JH. A systematic review and meta-analysis to revise the Fenton growth chart for preterm infants. *BMC Pediatrics*. 2013/04/20 2013;13(1):59. doi:10.1186/1471-2431-13-59

2. Battaglia FC, Lubchenco LO. A practical classification of newborn infants by weight and gestational age. *The Journal of Pediatrics*. 1967/08/01/ 1967;71(2):159-163. doi:<https://doi.org/10.1016/S0022-3476(67)80066-0>
