# Supplementary figures and images for: Using the PowerMom Digital Health Platform to Support Prenatal Mental Health and Maternal Health Outcomes: Observational Cohort Study
Source: JMIR Ment Health. 2025 May 22;12:e70151. doi: 10.2196/70151 (PMC12121545; doi:10.2196/70151)

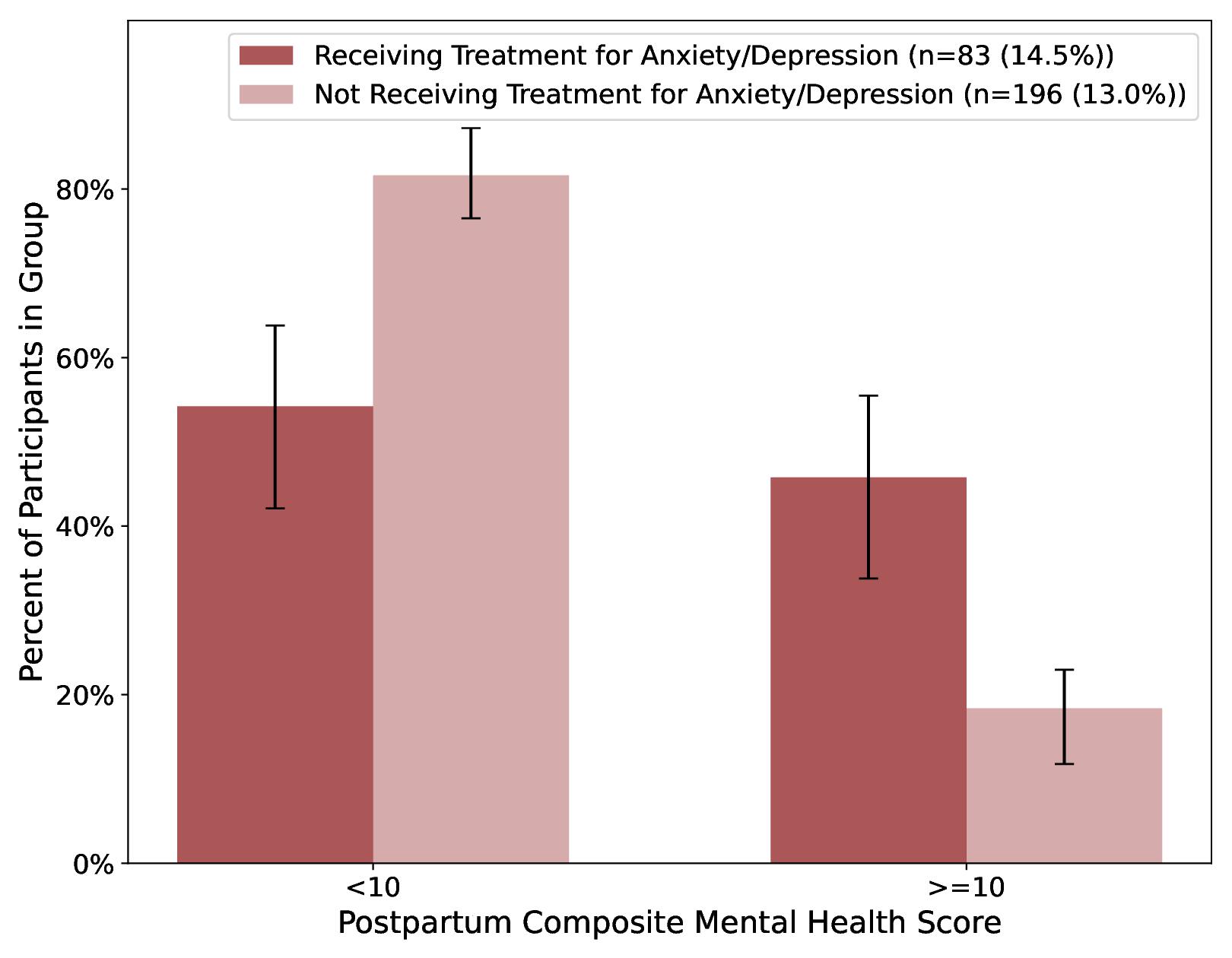

Supplement: Multimedia Appendix 2 [file mental-v12-e70151-s002.jpg]
